# Vision-Based Measurement of Heart Rate from Ballistocardiographic Head Movements Using Unsupervised Clustering

**DOI:** 10.3390/s19153263

**Published:** 2019-07-24

**Authors:** Hyunwoo Lee, Ayoung Cho, Seongwon Lee, Mincheol Whang

**Affiliations:** 1 Department of Emotion Engineering, University of Sangmyung, Seoul 03016, Korea; 2 Department of Intelligence Informatics Engineering, University of Sangmyung, Seoul 03016, Korea

**Keywords:** heart rate measurement, head movement, ballistocardiography, remote BCG, remote PPG, unsupervised clustering, k-means, camera

## Abstract

Heart rate has been measured comfortably using a camera without the skin-contact by the development of vision-based measurement. Despite the potential of the vision-based measurement, it has still presented limited ability due to the noise of illumination variance and motion artifacts. Remote ballistocardiography (BCG) was used to estimate heart rate from the ballistocardiographic head movements generated by the flow of blood through the carotid arteries. It was robust to illumination variance but still limited in the motion artifacts such as facial expressions and voluntary head motions. Recent studies on remote BCG focus on the improvement of signal extraction by minimizing the motion artifacts. They simply estimated the heart rate from the cardiac signal using peak detection and fast fourier transform (FFT). However, the heart rate estimation based on peak detection and FFT depend on the robust signal estimation. Thus, if the cardiac signal is contaminated with some noise, the heart rate cannot be estimated accurately. This study aimed to develop a novel method to improve heart rate estimation from ballistocardiographic head movements using the unsupervised clustering. First, the ballistocardiographic head movements were measured from facial video by detecting facial points using the good-feature-to-track (GFTT) algorithm and by tracking using the Kanade–Lucas–Tomasi (KLT) tracker. Second, the cardiac signal was extracted from the ballistocardiographic head movements by bandpass filter and principal component analysis (PCA). The relative power density (RPD) was extracted from its power spectrum between 0.75 Hz and 2.5 Hz. Third, the unsupervised clustering was performed to construct a model to estimate the heart rate from the RPD using the dataset consisting of the RPD and the heart rate measured from electrocardiogram (ECG). Finally, the heart rate was estimated from the RPD using the model. The proposed method was verified by comparing it with previous methods using the peak detection and the FFT. As a result, the proposed method estimated a more accurate heart rate than previous methods in three experiments by levels of the motion artifacts consisting of facial expressions and voluntary head motions. The four main contributions are as follows: (1) the unsupervised clustering improved the heart rate estimation by overcoming the motion artifacts (i.e., facial expressions and voluntary head motions); (2) the proposed method was verified by comparing with the previous methods using the peak detection and the FFT; (3) the proposed method can be combined with existing vision-based measurement and can improve their performance; (4) the proposed method was tested by three experiments considering the realistic environment including the motion artifacts, thus, it increases the possibility of the non-contact measurement in daily life.

## 1. Introduction

Heart rate is an important and popular indicator to monitor cardiac activity. It was traditionally measured by skin-contact sensors such as electrocardiography (ECG), photoplethysmography (PPG), and ballistocardiography (BCG). Although they are accurate methods of standardized measurement, it is often difficult to wear for a long period of time in order to avoid inconvenience, measurement burden, and skin damages [1]. Since the vision technology is advanced, the heart rate has been measured from facial videos without the skin-contact. It allows the possibility of the vision-based measurement of heart rate in daily life by reducing the measurement burden.

Despite the potential of the vision-based measurement, it has still presented limited ability to be applied in daily life. The main issues regarding the vision-based measurement are to improve signal-to-noise ratio (SNR) and to overcome the noise of illumination variance and motion artifacts [2]. The vision-based studies on the heart rate measurement have two concepts, i.e., PPG and BCG. Initial studies focused on remote PPG which measures the reflectance of blood due to the cardiac cycle from the heart to the head through the carotid arteries [1]. They captured subjects’ faces from videos and then recovered the plethysmographic signal from the RGB spectrum using several signal processing. Poh et al. [3] first proposed that the PPG signal can be extracted from the spatial mean of each of the R, G, B spectrums using independent component analysis (ICA). De Hann et al. developed more robust methods to the motion artifacts using chrominance features from the RGB spectrum [4] and using the Hulsbusch noise-free spectrum model [5]. Yan et al. [6] proposed an enhanced method to maximize the SNR by taking the weighted average of each color component of the RGB spectrum. Although the remote PPG has improved the issues of the SNR and motion artifacts, they have still presented limited ability to be applied in daily life due to the sensitivity for illumination variance.

Recent studies have investigated remote BCG measured by the ballistocardiographic head movements generated by the contraction of the heart and the ejection of the blood from ventricles into the vasculature [7]. They captured subjects’ faces from videos continuously and then calculated the ballistocardiographic head movements from the previous and next faces. The remote BCG can be subdivided into three steps as follows [8]: (1) signal extraction, (2) signal estimation, and (3) heart rate estimation. Signal extraction is to track the face from the facial videos and to extract the raw signal. Signal estimation is to estimate the ballistocardiographic signal from the raw signal by the noise cancellation. Heart rate estimation is to estimate the heart rate from the ballistocardiographic signal. Balakrishnan et al. [9] first proposed that the BCG signal can be extracted by tracking movements of facial points in 2-D trajectories. They cropped the regions of the face above the eye line and around the cheeks and the upper portion of the lips for improvement of the SNR, and then used principal component analysis (PCA) to eliminate the motion artifacts by the extract periodic signal. The main contribution of the remote BCG was invariant to illumination variance and variations of skin complexion, which was a hard issue in the remote PPG. However, they were still limited in the realistic environment including the motion artifacts such as facial expressions and voluntary head motions [2].

To overcome the limitation of the motion artifacts, previous studies have focused on how to improve the signal extraction and the signal estimation. Shan et al. [10] extract the raw signal by cropping only the forehead region instead of multiple regions, because the forehead region contains fewer voluntary movements than the other regions. Then, they applied a bandpass filter and ICA on the raw signal for motion artifacts removal. Haque et al. [11] developed a fusion method of corner feature points of good features to track (GFTT) and 49 facial points of a deformable face fitting algorithm. It improved the limitation of the GFTT-based tracking which is that the tracking is difficult due to the skin surface without the corner. Hassan et al. [12] developed robust face tracking by the skin color-based foreground segmentation method and enhanced the motion artifact removal by utilizing singular value decomposition (SVD) instead of the original PCA. Their methods are summarized as shown in Table 1. Although the signal extraction and the signal estimation have been improved by advanced tracking and filtering algorithms, the heart rate estimation is still based on traditional peak detection and fast fourier transform (FFT).

As shown in Table 1, the heart rate estimation generally has two concepts, i.e., (1) peak detection and (2) FFT [8]. In the method using the peak detection, the peaks were identified by the higher amplitude than its surroundings. Then, the peak-to-peak intervals (PPIs) were calculated as the time difference between the peak and the next peak. Although it is a standardized method to calculate heart rate, it was difficult to employ in a noisy environment due to its sensitivity to the shape of the signal. In the method using the FFT, the signal was transformed to the frequency domain by the FFT. The dominant frequency was identified with the highest power in the frequency domain and related to the PPI since the frequency shows the number of occurrences of repeating peaks. The heart rate was calculated by multiplying the dominant frequency by 60. Although it is less sensitive to the shape of the signal, the dominant frequency is vulnerable to the repetitive noise, such as the motion artifacts. The heart rate estimation based on the peak detection and the FFT depends on the advanced signal extraction and signal estimation, thus, it is necessary for developing the robust heart rate estimation to the noisy signal.

Machine learning approaches have been developed to improve the traditional signal processing such as the peak detection and the FFT. They learn by finding hidden features from the training data. Recently, deep learning approaches have been studied by corresponding the structure of the human brain. Especially, convolutional neural networks (CNNs) have achieved state-of-the-art performance in visual classification and time-series problems [13,14,15,16,17]. Indeed, a recent study on BCG showed that CNNs improved heart rate estimation by overcoming the limits of traditional signal processing [18]. However, it is difficult to interpret how CNNs show excellent performance by extracting hidden features from data. On the other hand, unsupervised clustering can extract common rules by clustering similar data, so that it is possible to interpret what signal corresponds to the heart rate [19]. This study hypothesized that the unsupervised clustering can extract the common rules for heart rate estimation and perform better than previous methods, such as the peak detection and the FFT.

This study aimed to develop a vision-based method to measure the heart rate from ballistocardiographic head movements using the unsupervised clustering. Facial videos were taken from three experiments by levels of the motion artifacts as follows: (1) normal, (2) facial expressions, and (3) facial expressions and voluntary head motions. The contribution of this study can be summarized as follows: (1) the unsupervised clustering improved the heart rate estimation by overcoming the motion artifacts (i.e., facial expressions and voluntary head motions); (2) the proposed method was verified by comparing with the previous methods using the peak detection and the FFT; (3) the proposed method can be combined with existing vision-based measurement and can improve their performance; (4) the proposed method was tested by three experiments considering the realistic environment including the motion artifacts, thus, it increases the possibility of the non-contact measurement in daily life.

## 2. Proposed Method

### 2.1. Signal Extraction and Signal Estimation

The cardiac signal was estimated from facial videos by measuring the ballistocardiographic head movements and filtering the noise. The signal extraction and the signal estimation of the mentioned remote BCG [9,10,11,12] can be employed in this step. To demonstrate the improvement of the proposed heart rate estimation by minimizing the effect of signal extraction and signal estimation, the initial remote BCG proposed by [9] was employed to estimate the cardiac signal in this study.

**Figure 1:**
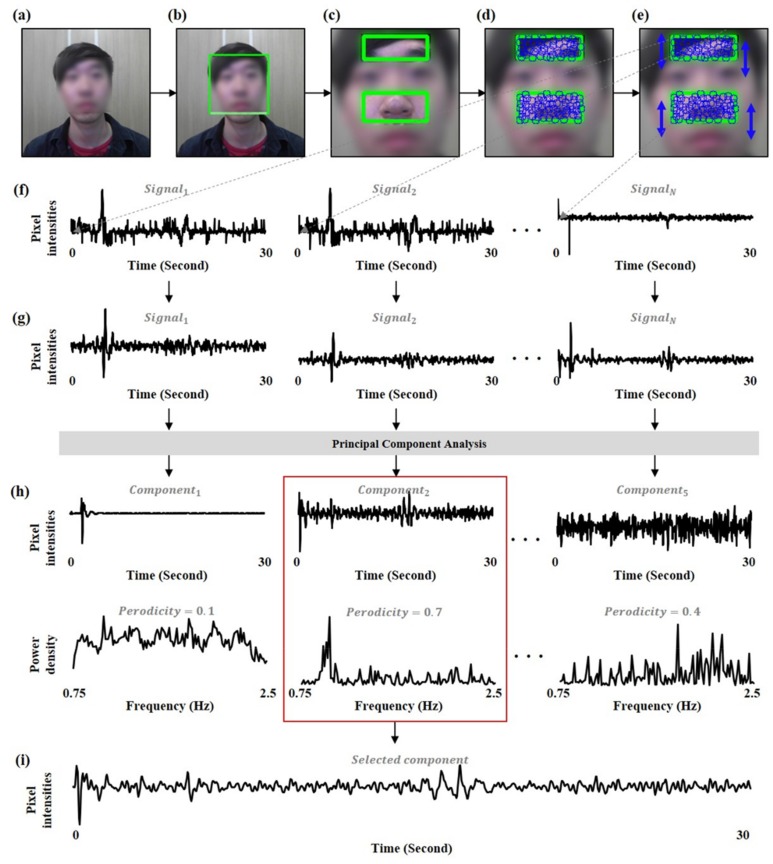
Overview of signal extraction and signal estimation. (**a**) facial video, (**b**) face detection, (**c**) area selection, (**d**) facial point extraction, (**e**) facial point tracking, (**f**) signal extraction using sliding window of 30 s, (**g**) bandpass filtering, (**h**) principal component analysis, (**i**) component selection with highest periodicity.

Figure 1 depicts the procedure of signal extraction and signal estimation. The first step of the signal extraction was face detection from facial video. The Viola–Jones face detector [20] was employed to extract the bounding box from the facial video. The bounding box was divided into the sub-bounding boxes by cropping on the middle 50% of the widthwise and top 20% of the heightwise (i.e., forehead) and on the middle 50% of the widthwise and middle 25% of heightwise (i.e., nose). The forehead and nose are more robust to facial expressions than other facial regions. Then, the facial points were determined within the sub-bounding boxes by the GFTT algorithm [21]. Because the ballistocardiographic head movements were generated up and down by the heartbeat, the raw signals were extracted by tracking the y-coordinate of each facial point using the Kanade–Lucas–Tomasi (KLT) tracker [22]. The raw signals were filtered by a 2nd order Butterworth bandpass filter with a cut-off of 0.75–2.5 Hz corresponding to 45–150 bpm. Finally, the PCA was performed to estimate the cardiac signal from the filtered signals. This study extracted 5 components by the PCA and then selected one component with the highest periodicity as the cardiac signal. Because the heartbeat is generated with harmonic components, the periodicity is an important feature to identify cardiac signal [9]. The periodicity was calculated from the frequency domain of each component as:

(1) $$Periodicitiy\frac{\underset{f}{\max}PS\left(f\right)}{\sum_{f=0.75}^{2.5}PS\left(f\right)}$$

where, Periodicitiy is a periodicity of each component, $PS\left(f\right)$ is a spectral density function of each component, $\underset{f}{max}PS\left(f\right)$ is a maximum spectral density of each component, and f is a frequency Hz.

### 2.2. Feature Extraction

To perform the unsupervised clustering, it is necessary to extract the features from the cardiac signal. This study extracted the features from the frequency domain by considering the harmonic component of the heartbeat. First, the cardiac signal was transformed to the frequency domain from the time domain by the FFT. Then, the power density was calculated from the frequency domain between 0.75 Hz and 2.5 Hz corresponding to the normal heartbeat. Finally, the relative power density (RPD) was extracted by the normalization as:

(2) $$TP=\overset{2.5}{\underset{f=0.75}{\sum }}PS\left(f\right)$$

(3) $$Norm\left(PS\left(f\right)\right)=\frac{PS\left(f\right)}{TP}$$

(4) $$RPD=\{Norm\left(PS\left(f\right)\right)|PS\left(f\right),f=0.75,0.75+df,0.75+2df,\ldots ,2.5\}$$

(5) $$df=\frac{fs}{len\left(PS\right)}$$

where, $PS\left(f\right)$ is a spectral density function of each component, f is a frequency Hz, TP is a total power of the power spectrum between 0.75 Hz and 2.5 Hz, Norm is a normalization, df is a frequency resolution, fs is a sampling rate, and $len\left(PS\right)$ is a length of the power spectrum. This study determined the RPD was the features for the unsupervised clustering. If the signal was measured at a sampling rate of 30 Hz for 30 s, df is determined as 0.033 by calculating $\frac{30}{900}$, so that the RPD has 53 dimensions by calculating $\frac{2.5-0.75}{0.033}$.

**Figure 2:**
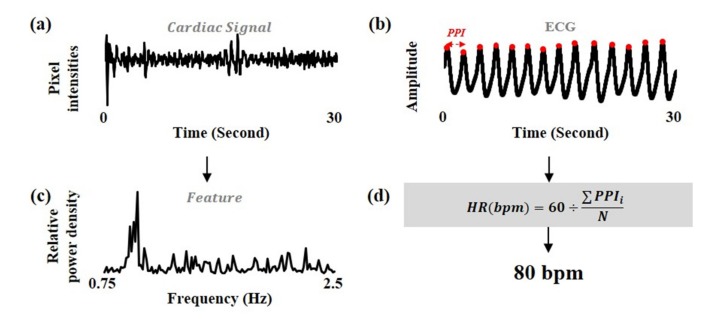
Overview of feature extraction. (**a**) cardiac signal estimated from facial video by signal extraction and signal estimation, (**b**) electrocardiogram (ECG) signal, (**c**) relative power density (RPD) extracted from the cardiac signal by fast fourier transform (FFT) and normalization and determined as features, (**d**) heart rate calculated from the ECG signal by the QRS detection algorithm and determined as labels.

The labels corresponding to the features were extracted from the synchronized ECG signals. The 2nd order Butterworth bandpass filter with the cut-off frequencies of 0.75–2.5 Hz was applied on the ECG signals. The heart rate was calculated from the signals by the QRS detection algorithm, which was implemented by Pan and Tompkins to detect the peaks by considering the QRS complex in ECG signal [23]. The datasets were constructed using the RPD as the features and the heart rate of ECG signals as the labels. Figure 2 shows the procedure of feature extraction.

### 2.3. Unsupervised Clustering

**Figure 3:**
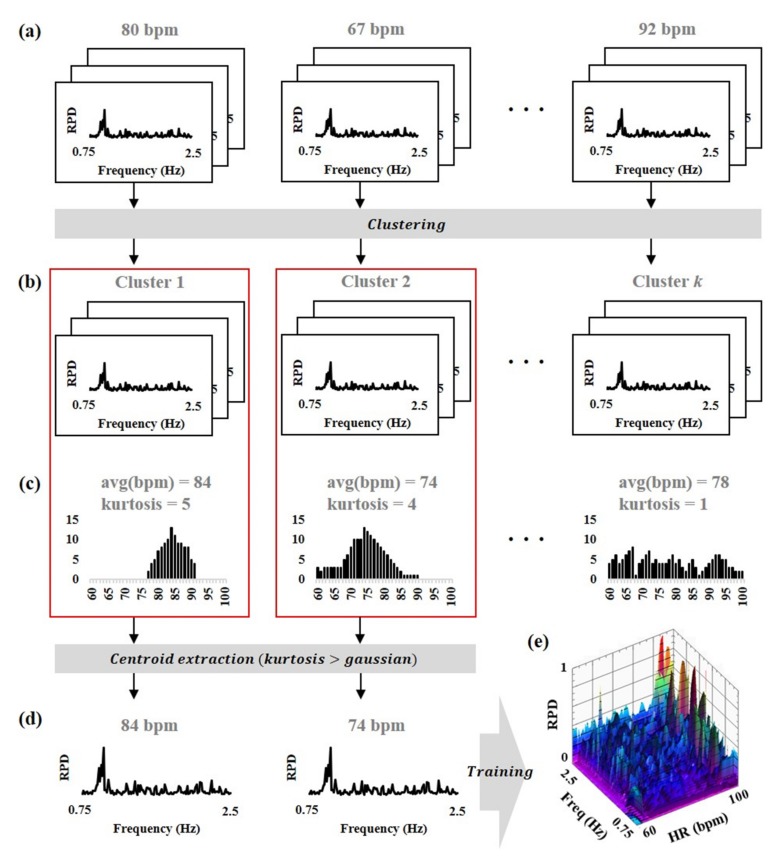
Overview of training model using the unsupervised clustering. (**a**) Dataset consisting of RPD (i.e., features) and heart rate (i.e., labels), (**b**) unsupervised clustering with *k* clusters, (**c**) kurtosis of distribution calculated from each cluster, (**d**) centroid extraction of each cluster with higher kurtosis than gaussian, (**e**) trained model consisting of RPD and heart rate.

The unsupervised clustering was performed to train a model to estimate the heart rate using the dataset consisting of the features and its labels, as shown in Figure 3. This study employed k-means clustering [24] by minimizing the Euclidean distance between the features and their centroid as:

(6) $$UD\left(p,q\right)=∥p-q{∥}^{2}$$

(7) $$arg\underset{C}{\min}\overset{n}{\underset{i=1}{\sum }}\underset{x\in C_{i}}{\sum }UD\left(x,u_{i}\right)$$

where, UD is a Euclidean distance, p and q are two points in *d*-dimension, C is a cluster, $u_{i}$ is a centroid of the cluster $C_{i}$, x is a *d*-dimensional real vector, n is a number of clusters which is a hyper-parameter-determined by the researcher, and i is an index of each cluster. In this study, the RPD was determined as x. Equation (7) is the optimization function to determine members of each cluster. Because the number of clusters (i.e., *n*) was not determined initially, the candidates were extracted by performing the clustering while increasing *n* from 3 to *N*, where *N* is the number of non-duplicate labels in the dataset. If the kurtosis of each cluster was larger than the one of the gaussian distribution, the candidate was registered into the model.

### 2.4. Heart Rate Estimation

**Figure 4:**
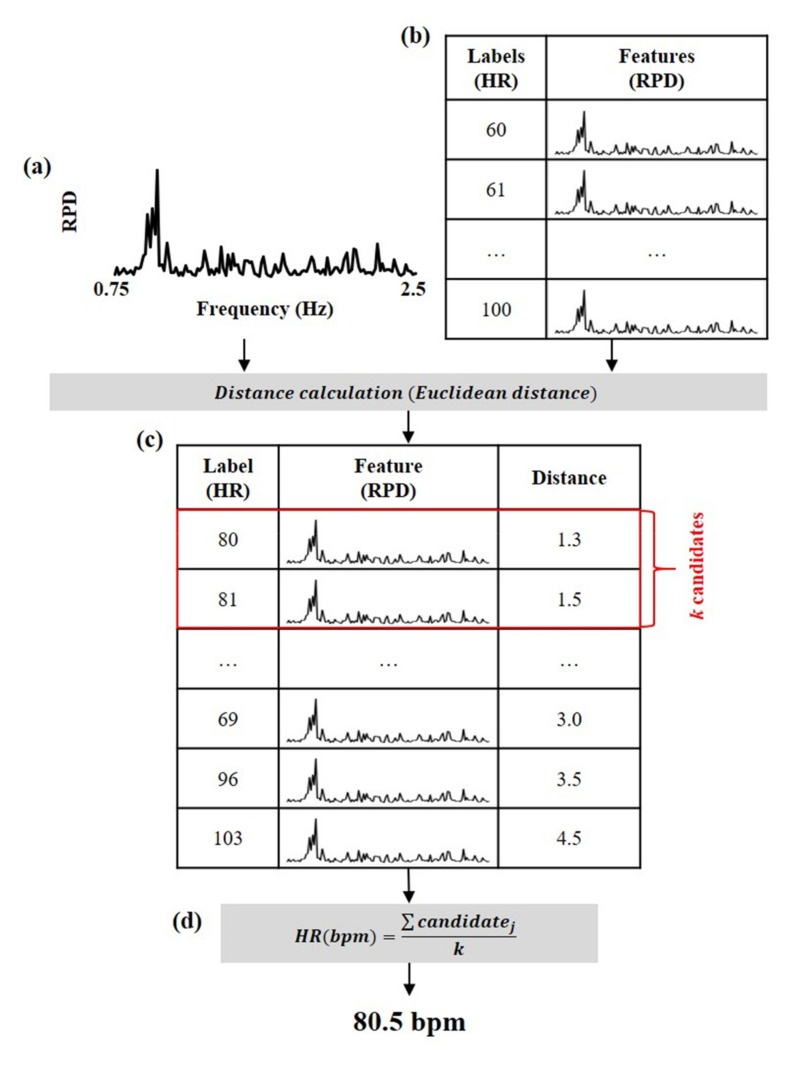
Overview of heart rate estimation using the model. (**a**) The RPD extracted from the facial video, (**b**) the trained model consisting of RPD and heart rate, (**c**) distance calculation between the RPD of the facial video and the RPDs of the model, (**d**) heart rate estimation by averaging the *k* candidates.

Heart rate was estimated from the RPD using the model, as shown in Figure 4. The model consists of the RPDs (i.e., features) and corresponding heart rates (i.e., labels). First, the Euclidean distances between the RPD of the facial video and the RPDs of the model were calculated. Then, *k* candidates were extracted in descending order of the distance. The heart rate was finally estimated by averaging the *k* candidates. Since the optimized *k* parameter was not known, it was determined by minimizing the differences between labels and estimates in experiments as:

(8) $$argmin_{k}\frac{\sum_{j=1}^{k}\left|label_{j}-estimate_{j}\right|}{k}$$

where, k is an optimized number of candidates for heart rate estimation, and j is an index of each candidate.

## 3. Experiments

### 3.1. Dataset

This study evaluated the proposed method from three experiments according to measurement conditions. The measurement conditions were determined by levels of the motion artifacts as follows: (1) normal, (2) facial expressions, and (3) facial expressions and voluntary head motions.

**Experiment 1: normal.** This experiment was to collect the normal dataset without facial expressions and voluntary head motions. The participants consisted of twenty persons (12 males). They were asked to sit in front of a camera for 3 min with a stationary state and to keep a neutral facial expression. The facial video was recorded by an RGB webcam (Logitech Webcam C270) at 30 fps and 640 × 360 pixels. The webcam was installed 1 m away from the participant. The ECG signal was simultaneously measured at a sampling rate of 500 Hz by an ECG measurement system with Lead-I (BIOPAC Systems Inc., Goleta, CA, USA). It served as a ground-truth for the evaluation of our method. This experiment was approved by the Institutional Review Board of the Sangmyung University, Seoul, Korea (BE2018-35).

**Experiment 2: facial expressions.** This experiment was to collect the dataset including the motion artifacts by facial expressions. The participants consisted of twenty persons (12 males) who also participated in experiment 1. They were asked to sit in front of a camera for 3 min with a stationary state and to follow the six facial expressions (i.e., happiness, sadness, surprise, anger, disgust, and fear) on the monitor. Each facial expression was displayed on the monitor for 30 s in random order to minimize the ordering effect. The facial video was recorded by the webcam used in experiment 1. The ECG signal was simultaneously measured at a sampling rate of 500 Hz by an ECG measurement system with Lead-I (BIOPAC Systems Inc., USA) for the ground-truth. This experiment was approved by the Institutional Review Board of the Sangmyung University, Seoul, Korea (BE2018-35).

**Experiment 3: facial expressions and voluntary head motions.** This experiment was to collect the dataset including the motion artifacts by facial expressions and voluntary head motions. This study employed the publicly available MAHNOB-HCI database [25]. The participants consisted of 30 persons (13 males) aged 26.06 ± 4.39 years. They were asked to freely express their facial expressions and move their heads while watching a video which induced emotions. The facial video was recorded by a RGB webcam (Allied Vision Stingray F-046C) at 60 fps and 780 × 580 pixels. The ECG signal was simultaneously measured at a sampling rate of 256 Hz by a Biosemi active II system with active electrodes for the ground-truth. This study tested the data from 26 participants, excluding 4 participants due to technical problems and unfinished data collection.

**Figure 5:**
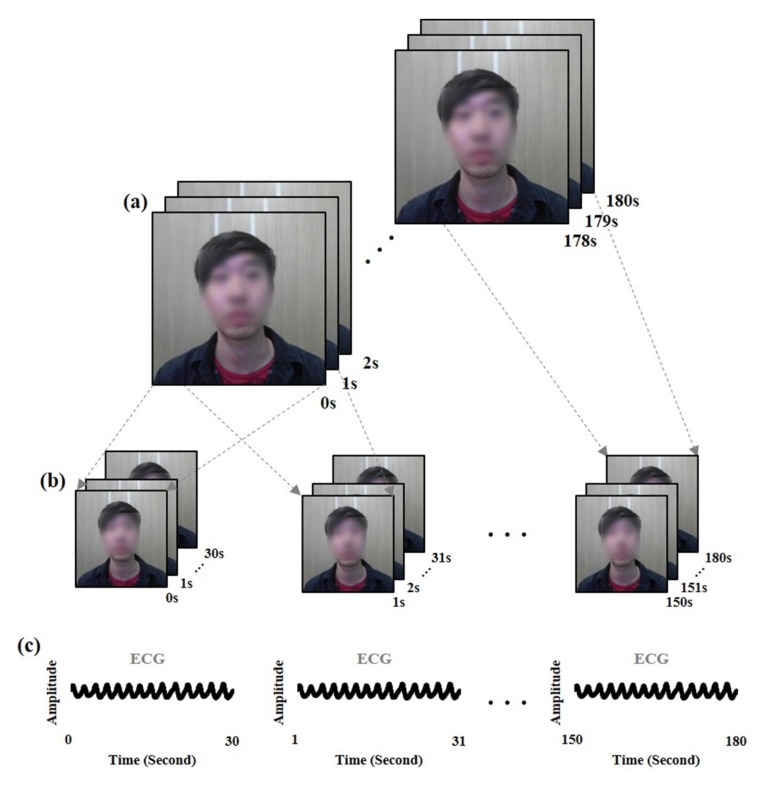
Overview of signal segmentation by sliding window. (**a**) Facial video recorded for 3 min, (**b**) facial video segmented by sliding window (window size = 30 s, interval size = 1 s), (**c**) ECG signal corresponding to the segmented facial video.

The facial video and corresponding ECG signal were segmented as a sliding window (window size = 30 s, interval size = 1 s), as shown in Figure 5. The samples were divided into training and test datasets for cross-validation. For instance, if the samples from participant 1 were tested, the samples from other participants were determined as the training dataset. Furthermore, the samples of the same participant were not included in both training and test datasets. The training and test datasets were employed to construct a model by unsupervised clustering and to evaluate the method, respectively. In experiments 1 and 2, the size of the dataset from one participant was 150 samples due to their facial video, which was recorded for 3 min. Thus, the training and test datasets included 2,850 and 150 samples, respectively. In experiment 3, the size of the dataset was not fixed due to the recording time of the facial video, which differed from participant to participant. On average, the training and test datasets included 6,250 and 50 samples, respectively.

### 3.2. Evaluation

As described in Section 2.4, the *k* parameter about the number of candidates for heart rate estimation should be optimized by the experiments. A small *k* parameter can track instantaneous changes of heart rate, but it is also sensitive to slight variations and can be unstable. On the other hand, a large *k* parameter is smoothed and stable, similar to the effect of the moving averaging, but it cannot reflect instantaneous changes of heart rate. Thus, it is important to determine the *k* parameter by optimization of this trade-off. This study tested the differences between labels and estimates according to the *k* parameter from 1 to 10 in each dataset. The *k* parameter was determined when the difference was minimum.

**Figure 6:**
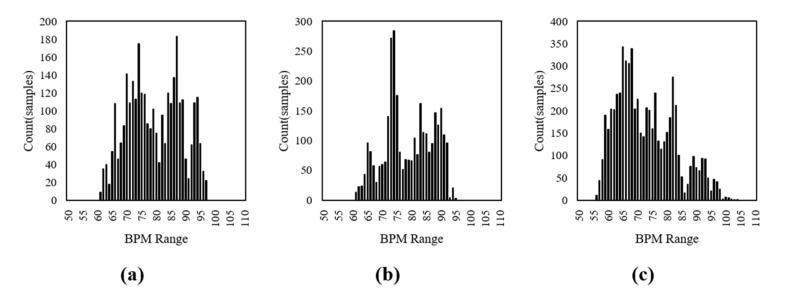
Distributions of the dataset extracted from the three experiments. (**a**) Experiment 1: normal, (**b**) experiment 2: facial expressions, (**c**) experiment 3: facial expressions and voluntary head motions.

**Figure 7:**
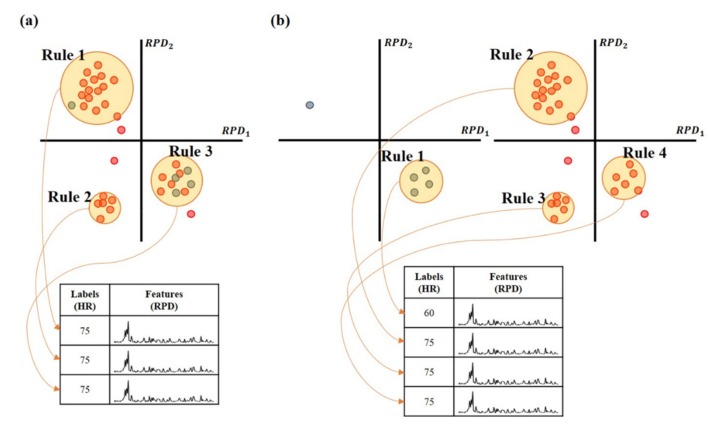
Example of model training by dividing the training dataset into 10 bpm units and by combining their models. In this example, the dataset consisted of 5 samples for 60 bpm (i.e., blue circle) and 30 samples for 75 bpm (i.e., red circle). Assume that their RPD has 2 dimensions to facilitate the visualization. (**a**) The clustering was performed on the entire dataset, so that the rule for 60 bpm was not trained from the imbalanced dataset. (**b**) The clustering was performed by dividing the training dataset into 10 bpm units, so that the rule for 60 bpm can be trained from the divided dataset.

Figure 6 shows the distributions of the datasets extracted from the three experiments. They have an imbalance problem induced by the overfitting for the training model. So, this study divided the training dataset into 10 bpm units (i.e., 50~60 bpm, 60~70 bpm, …, 90~100 bpm) and then performed the clustering for each unit to solve the imbalance problem. The final model was determined by combining the candidate models trained from each clustering, as shown in Figure 7.

The contribution of the proposed method was to develop an enhanced heart rate estimation by using unsupervised clustering. This study verified the enhancement by comparing the proposed method with previous methods using peak detection [26] and FFT [9]. They were evaluated by calculating the metrics based on mean absolute error (MAE), standard deviation of absolute error (SDAE), root mean squared error (RMSE), Pearson’s correlation coefficient (CC), and the Bland–Altman plot. MAE, SDAE, and RMSE quantifies the difference of average heart rate and variation, respectively. CC quantifies the similarity of heart rates accumulated over a time window. The coefficient value was indicated as a strong positive similarity if it was approaching the 1 and as statistically significant if the *p*-value was less than 0.05. All statistical values were calculated with beat-to-beat heart rate, not average heart rate, for a more rigorous evaluation. The Bland–Altman plot was to interpret the differences between other methods graphically and statistically. The plot was represented by assigning the mean (x-axis) and difference (y-axis) between the two measurements. The 95% limits of an agreement were calculated by mean difference and the 1.96 standard deviation of the difference was represented as lines on the plot.

## 4. Results

### 4.1. Experiment 1: Normal

**Figure 8:**
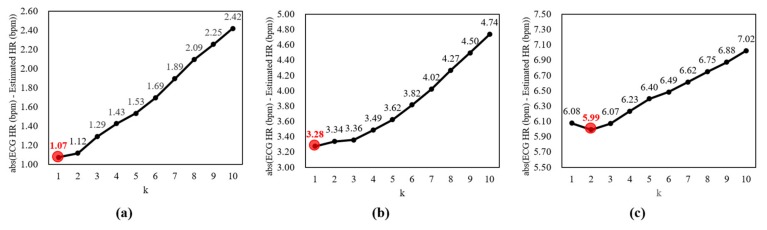
Differences between labels and estimates according to *k* parameter for heart rate estimation. (**a**) Normal dataset without facial expressions and voluntary head motions, (**b**) dataset with facial expressions, (**c**) dataset with facial expressions and voluntary head motions.

Figure 8a shows the differences between labels and estimates according to the *k* parameter for heart rate estimation in the normal dataset without facial expressions and voluntary head motions. The differences decrease until the *k* parameter is 1 (i.e., 1.07 bpm), and then increase continuously until the *k* parameter is 10 (i.e., 2.42 bpm). Thus, the *k* parameter was defined as 1, which shows the lowest difference.

The heart rates estimated using peak detection, FFT, and clustering were evaluated with respected analyses. Table 2 shows the estimated heart rates from the normal dataset without facial expressions and voluntary head motions. The errors of the normal dataset were lowest when the clustering was employed (MAE = 1.07, SDAE = 0.99, RMSE = 1.47, CC = 0.999).

**Figure 9:**
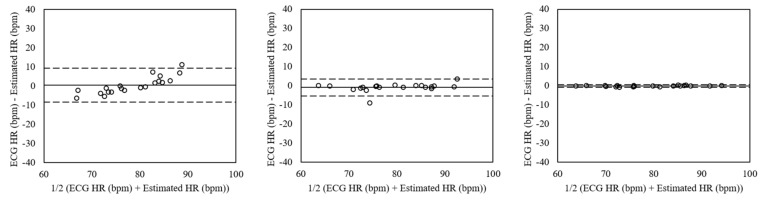
Bland–Altman plots of heart rates estimated from the normal dataset without facial expressions and voluntary head motions using peak detection (**Left**), FFT (**Mid**), and clustering (**Right**). The lines are the mean errors and 95% LOA.

The Bland–Altman plots of estimated heart rates from the normal dataset without facial expressions and voluntary head motions using peak detection, FFT, and clustering are shown in Figure 9. The mean errors were 0.46 with 95% limits of agreement (LOA) in −8.36 to 9.28 (peak detection), −0.86 with 95% LOA in −5.25 to 3.54 (FFT), and 0.13 with 95% LOA in −0.75 to 0.51 (clustering). The differences of the heart rates were lower using clustering than the ones using peak detection and FFT, which showed that the hearts rates were almost synchronized.

### 4.2. Experiment 2: Facial Expressions

Figure 8b shows the differences between labels and estimates according to the *k* parameter for heart rate estimation in the dataset with facial expressions. Similar to the result of the normal dataset, the difference was lowest when the *k* parameter was 1 (i.e., 3.28 bpm). Therefore, it was determined as 1.

The heart rates were estimated from the dataset including the motion artifacts by facial expressions using peak detection, FFT, and clustering, as shown in Table 3. The errors were also lower using clustering than using peak detection and FFT (MAE = 3.28, SDAE = 3.45, RMSE = 4.48, CC = 0.970).

**Figure 10:**
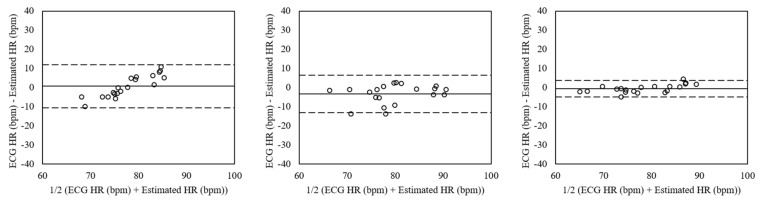
Bland–Altman plots of heart rates estimated from the dataset including the motion artifacts by facial expressions using peak detection (**Left**), FFT (**Mid**), and clustering (**Right**). The lines are the mean errors and 95% LOA.

Figure 10 shows the Bland–Altman plots of heart rates estimated from the dataset including the motion artifacts by facial expressions using peak detection, FFT, and clustering. The mean errors were 0.72 with 95% LOA in −10.52 to 11.96 (peak detection), −3.25 with 95% LOA in –13.03 to 6.54 (FFT), and −0.17 with 95% LOA in −4.82 to 3.77 (clustering). The heart rates estimated using clustering showed the lowest differences.

### 4.3. Experiment 3: Facial Expressions and Voluntary Head Motions

Figure 8c shows the differences between labels and estimates according to the *k* parameter for heart rate estimation in the dataset with facial expressions and voluntary head motions. The differences decrease until the *k* parameter is 2 (i.e., 5.99 bpm), and then increase continuously until the *k* parameter is 10 (i.e., 7.02 bpm). Thus, the *k* parameter was determined as 2 that shows the lowest difference.

Table 4 presents the heart rates evaluated from the dataset including the motion artifacts by facial expressions and voluntary head motions using peak detection, FFT, and clustering. The errors of the dataset were lowest when the clustering was employed (MAE = 5.99, SDAE = 5.24, RMSE = 8.09, CC = 0.836).

**Figure 11:**
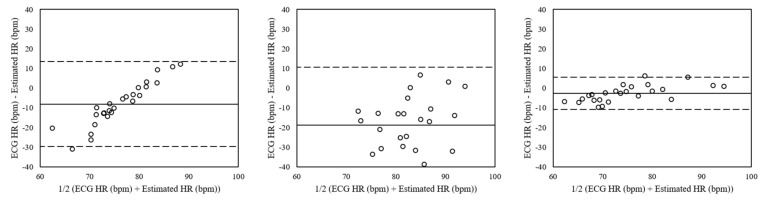
Bland–Altman plots of heart rates estimated from the dataset including the motion artifacts by facial expressions and voluntary head motions using peak detection (**Left**), FFT (**Mid**), and clustering (**Right**). The lines are the mean errors and 95% LOA.

The Bland–Altman plots of estimated heart rates from the dataset including the motion artifacts by facial expressions and voluntary head motions using each estimation method are shown in Figure 11. The mean errors were −8.05 with 95% LOA in −29.62 to 13.51 (peak detection), −18.75 with 95% LOA in −48.16 to 10.65 (FFT), and −0.25 with 95% LOA in −10.75 to 5.62 (clustering). The differences of the heart rates were lower using clustering than the ones using peak detection and FFT. Note that the plots of the peak detection and the clustering seem to have a linear correlation between the error and the ground-truth, so that they are more suitable to be compared with each other using mean errors.

## 5. Discussion

In this study, the clustering-based method was developed to enhance the heart rate estimation by replacing the traditional peak detection and FFT-based methods. This study evaluated the proposed method on three datasets according to levels of the motion artifacts as follows: (1) normal, (2) facial expressions, and (3) facial expressions and voluntary head motions. The proposed method was better than the peak detection and FFT-based methods in all datasets.

Overall, this study has drawn three significant findings. First, this study focused on the development of the heart rate estimation from the cardiac signal estimated from facial videos. The proposed method was demonstrated by comparing it with the peak detection and FFT-based heart rate estimation. In this demonstration, the cardiac signal was estimated using the remote BCG proposed by [9]. This method was first proposed by taking advantage of the ballistocardiographic head movements generated by the flow of blood through the carotid arteries. They showed the possibility of the remote BCG by demonstrating improved accuracy than the remote PPG in a noisy environment including illumination variance. In addition, the remote BCG can further lower the complexity of the hardware because they do not need color images. However, it is difficult to extract only the cardiac signal from the various head movements, including facial expressions and head motions, so that they were limited to overcome the motion artifacts. Recently, the improved methods based on the remote BCG have been developed by minimizing the motion artifacts and extracting the invariant features [10,11,12]. If the cardiac signal is estimated using the improved methods and then the heart rate is estimated using the proposed method in this study, it is expected to improve the accuracy in a realistic environment.

Second, the proposed method is a machine learning ML-based method using the unsupervised clustering. It is important to collect the dataset for training the model in the ML-based method. Its performance can be improved if the invariant features are extracted from the dataset, including various noise. In vision-based measurement studies, the illumination variance and motion artifacts are considered as major noise sources. Thus, it is necessary to collect more datasets, including the noise due to the illumination variance and motion artifacts. If the model is constructed with more collected datasets, the accuracy can be improved in a noisy environment.

Third, Table 5 shows the comparison of the proposed method and other remote BCG methods in experiment 3, where an online database was used [9,10,11,12]. The compared methods were evaluated by calculating MAE, SDAE, RMSE, and CC in [12]. The proposed method presented lower errors than the other three methods [9,10,11], whereas it showed higher errors than the most recently developed method [12] using the improved signal extraction and signal estimation. It indicated that the signal extraction and signal estimation should be improved as well as the heart rate estimation to overcome the motion artifacts for remote BCG. Note that the proposed method showed lower variance of errors (i.e., SDAE = 5.24) than the previous method [9] using the same signal extraction and signal estimation (i.e., SDAE = 11.91). This comparison reflects only the improved heart rate estimation, except for the signal extraction and signal estimation. It indicated that the proposed method using the unsupervised clustering can estimate heart rate with more stability than other methods using the peak detection and the FFT. Thus, this study is expected to demonstrate higher performance if the heart rate is estimated by the proposed heart rate estimation using the unsupervised clustering from the cardiac signal extracted by the improved signal extraction and signal estimation.

This study explored the issues regarding the remote BCG in the measurement conditions according to the motion artifacts as follows: (1) normal, (2) facial expressions, and (3) facial expressions and voluntary head motions. First, the possibility of overcoming the motion artifacts for the remote BCG was shown by the development of the clustering-based method. Second, the proposed method was demonstrated to better reduce the motion artifacts than traditional peak detection and FFT-based methods and to more accurately estimate the heart rate. Third, the possibility of combining it with existing vision-based measurements and improving their performance. Consequently, our findings represent a significant step towards ensuring the enhanced development of remote BCG.

## 6. Conclusions

This study estimated the heart rate from facial videos using remote BCG based on unsupervised clustering. The proposed method using the clustering was compared with the previous heart rate estimation using peak detection and FFT. As a result, the proposed method estimated a more accurate heart rate than peak detection and FFT in three experiment datasets with difference levels of motion artifacts. Specifically, it demonstrated the ability to overcome the motion artifacts problem for remote BCG in both stable and unstable environments. Although it showed higher errors than some other remote BCG methods which improve the signal extraction and signal estimation, it is expected to overcome this issue if the heart rate is estimated by the proposed heart rate estimation from the cardiac signal extracted by their signal extraction and signal estimation. The findings are a significant step toward ensuring the enhanced development of remote BCG. This study is expected to help more accurately estimate the heart rate by overcoming the motion artifacts problem and consequently improving the measurement environment in daily life.

## Figures and Tables

**Table 1:** Summary of published remote ballistocardiography (BCG) studies.

Methods	Signal Extraction	Signal Estimation	Heart Rate Estimation
Bal et al. 2013 [9]	VJ + GFTT + KLT	Bandpass + PCA	Peak detection, FFT
Shan et al. 2013 [10]	VJ + GFTT + KLT	Norm + Bandpass + ICA	FFT
Haque et al. 2016 [11]	VJ + GFTT + SDM	Bandpass + MA + PCA	FFT
Hassan et al. 2017 [12]	VJ + SCFS + KLT	Bandpass + SVD	FFT

VJ = Viola-Jones algorithm; GFTT = good-features-to-track; KLT = Kanade–Lucas–Tomasi tracking algorithm; SDM = supervised descent method; SCFS = skin color-based foreground segmentation; Norm = normalization; MA = moving averaging; PCA = principal component analysis; ICA = independent component analysis; FFT = fast fourier transform.

**Table 2:** Estimation of heart rates from the normal dataset without facial expressions and voluntary head motions.

Heart Rate Estimation	MAE	SDAE	RMSE	CC
Peak Detection	3.95	2.49	4.70	0.933 **
FFT	2.76	5.91	6.61	0.967 **
Clustering	1.07	0.99	1.47	0.999 **

MAE = mean absolute error; SDAE = standard deviation of absolute error; RMSE = root mean square error; CC = Pearson’s correlation coefficient. Two asterisk represents significant correlation levels at *p*-value < 0.01. The lowest error and highest correlation values are bolded.

**Table 3:** Estimation of heart rates from the dataset including the motion artifacts by facial expressions.

Heart Rate Estimation	MAE	SDAE	RMSE	CC
Peak Detection	5.66	3.81	6.85	0.829 **
FFT	10.08	12.93	16.68	0.776 **
Clustering	3.28	3.45	4.84	0.970 **

MAE = mean absolute error; SDAE = standard deviation of absolute error; RMSE = root mean square error; CC = Pearson’s correlation coefficient. Two asterisk represents significant correlation levels at *p*-value < 0.01. The lowest error and highest correlation values are bolded.

**Table 4:** Estimation of heart rates from the dataset including the motion artifacts by facial expressions and voluntary head motions.

Heart Rate Estimation.	MAE	SDAE	RMSE	CC
Peak Detection	11.74	3.96	12.56	0.290
FFT	23.89	15.71	29.33	0.066
Clustering	5.99	5.24	8.09	0.836 **

MAE = mean absolute error; SDAE = standard deviation of absolute error; RMSE = root mean square error; CC = Pearson’s correlation coefficient. Two asterisk represents significant correlation levels at *p*-value < 0.01. The lowest error and highest correlation values are bolded.

**Table 5:** Comparison of the proposed method and other remote BCG methods in experiment 3.

Methods	MAE	SDAE	RMSE	CC
Bal et al. 2013 [9]	21.68	11.91	24.72	0.10
Shan et al. 2013 [10]	7.88	4.66	9.14	0.27
Haque et al. 2016 [11]	6.47	3.62	7.56	0.84**
Hassan et al. 2017 [12]	4.34	3.14	5.29	0.921**
Proposed method	5.99	5.24	8.09	0.836**

MAE = mean absolute error; SDAE = standard deviation of absolute error; RMSE = root mean square error; CC = Pearson’s correlation coefficient. Two asterisk represents significant correlation levels at *p*-value < 0.01. The lowest error and highest correlation values are bolded.
